# Supplementary material for: Prompt treatment of fever and its associated factors among under-five children in sub-Saharan Africa: A multilevel analysis of evidence from 36 countries
Source: PLoS One. 2024 May 16;19(5):e0303680. doi: 10.1371/journal.pone.0303680 (PMC11098344; doi:10.1371/journal.pone.0303680)
Supplement: Attachment — Submitted filename: PONE-D-24-00421.pdf [file pone.0303680.s001.pdf]

PLOS ONE

Prompt Treatment of Fever and its Associated Factors among Under-five Children in sub-Saharan Africa: A Multilevel Analysis of Evidence from 36 Countries

--Manuscript Draft--

**Manuscript Number:**	PONE-D-24-00421
**Article Type:**	Research Article
**Full Title:**	Prompt Treatment of Fever and its Associated Factors among Under-five Children in sub-Saharan Africa: A Multilevel Analysis of Evidence from 36 Countries
**Short Title:**	Prompt Treatment of Fever and its Associated Factors among Under-five Children in sub-Saharan Africa
**Corresponding Author:**	Enyew Getaneh Mekonen<br>University of Gondar College of Medicine and Health Sciences<br>Gondar, ETHIOPIA
**Keywords:**	Prompt treatment of fever<br>Under-five children<br>DHS<br>Sub-Saharan Africa<br>Multilevel analysis
**Abstract:**	Background: Despite the decrease in the global under-five mortality rate, the highest rates of mortality are reported in sub-Saharan Africa. More than one-third of all deaths among under-five children are either from lower respiratory tract infections, diarrhea, or malaria. Poor treatment-seeking behavior for fever among mothers of under-five children is a big concern in sub-Saharan Africa. However, the pooled prevalence of prompt treatment of fever and its associated factors among under-five children in the region using nationally representative data is not known. Therefore, the findings of this study will inform policymakers and program managers who work on child health to design interventions to improve the timely and appropriate treatment of fever among under-five children.<br>Methods: Data from the recent demographic and health surveys of 36 countries in sub-Saharan Africa conducted between 2006 and 2022 were used. A total weighted sample of 71,503 living children aged under five years with a fever was included in the study. Data extracted from DHS data sets were cleaned, recorded, and analyzed using STATA/SE version 14.0 statistical software. Multilevel mixed-effects logistic regression was used to determine the factors associated with the outcome variable. Intra-class correlation coefficient, likelihood ratio test, median odds ratio, and deviance (-2LLR) values were used for model comparison and fitness. Finally, variables with a p-value <0.05 and an adjusted odds ratio with a 95% confidence interval were declared statistically significant.<br>Results: The pooled prevalence of prompt treatment of fever among under-five children in sub-Saharan African countries was 26.11% (95% CI: 25.79%, 26.44%). Factors like maternal education [AOR = 1.18; 95% CI (1.13, 1.25)], maternal working status [AOR = 1.34; 95% CI (1.27, 1.41)], media exposure [AOR = 1.05; 95% CI (1.01, 1.10)], household wealth index [AOR = 1.13; 95% CI (1.06, 1.19)], distance to a health facility [AOR = 1.18; 95% CI (1.13, 1.23)], healthcare decisions [AOR = 1.34; 95% CI (1.01, 1.77)], visited healthcare facility last 12 months [AOR = 1.45; 95% CI (1.38, 1.52)], antenatal care attendance [AOR = 1.79; 95% CI (1.61, 1.99)], place of delivery [AOR = 1.55; 95% CI (1.47, 1.63)], and community-level antenatal care utilization [AOR = 1.08; 95% CI (1.02,1.14)] were significantly associated with prompt treatment of fever among under-five children.<br>Conclusion: The pooled prevalence of prompt treatment of fever among under-five children in sub-Saharan African countries was low. Educated women, working mothers, having media exposure, rich household wealth status, perceiving distance to a health facility was not a big problem, making healthcare decisions with husband or partner, visiting healthcare facility in the last 12 months, antenatal care attendance, health facility delivery, and high community-level antenatal care utilization increase the odds of prompt treatment of fever. Therefore, women’s empowerment, information dissemination through mass media, maintaining regular visits to healthcare facilities, and strengthening health facility delivery and antenatal care services are strongly

Powered by Editorial Manager® and ProduXion Manager® from Aries Systems Corporation

	recommended.
Order of Authors:	Enyew Getaneh Mekonen
	Belayneh Shetie Workneh
	Tadesse Tarik Tamir
	Alebachew Ferede Zegeye
Additional Information:	
**Question**	**Response**
**Financial Disclosure**<br><br>Enter a financial disclosure statement that describes the sources of funding for the work included in this submission. Review the submission guidelines for detailed requirements. View published research articles from *PLOS ONE* for specific examples.<br><br>This statement is required for submission and **will appear in the published article** if the submission is accepted. Please make sure it is accurate.<br><br>**Funded studies**<br>Enter a statement with the following details:<br>• Initials of the authors who received each award<br>• Grant numbers awarded to each author<br>• The full name of each funder<br>• URL of each funder website<br>• Did the sponsors or funders play any role in the study design, data collection and analysis, decision to publish, or preparation of the manuscript?<br><br>Did you receive funding for this work?	The author(s) received no specific funding for this work.
**Competing Interests**<br><br>Use the instructions below to enter a competing interest statement for this submission. On behalf of all authors, disclose any competing interests that could be perceived to bias this work—acknowledging all financial support and any other relevant financial or non-financial competing interests.	The authors have declared that no competing interests exist.

This statement is required for submission and **will appear in the published article** if the submission is accepted. Please make sure it is accurate and that any funding sources listed in your Funding Information later in the submission form are also declared in your Financial Disclosure statement.<br><br>View published research articles from *PLOS ONE* for specific examples.<br><br>**NO authors have competing interests**<br><br>Enter: *The authors have declared that no competing interests exist.*<br><br>**Authors with competing interests**<br><br>Enter competing interest details beginning with this statement:<br><br>*I have read the journal's policy and the authors of this manuscript have the following competing interests: [insert competing interests here]*<br><br>* typeset	
**Ethics Statement**<br><br>Enter an ethics statement for this submission. This statement is required if the study involved:<br><br>• Human participants<br>• Human specimens or tissue<br>• Vertebrate animals or cephalopods<br>• Vertebrate embryos or tissues<br>• Field research<br><br>Write "N/A" if the submission does not require an ethics statement.<br><br>General guidance is provided below. Consult the submission guidelines for detailed instructions. **Make sure that all information entered here is included in the**	Permission was granted to download and use the data from http://www.dhs.program.com before conducting the study. Ethical clearance was obtained from the Institution Review Board of the DHS Program, ICF International. The procedures for DHS public-use data sets were approved by the Institution Review Board. Identifiers for respondents, households, or sample communities were not allowed in any way, and the names of individuals or household addresses were not included in the data files. The number for each EA in the data file does not have labels to show their names or locations. There were no patients or members of the public involved since this study used a publicly available data set. As the study uses a retrospective study of archived samples, we have ensured that all data were fully anonymized before we accessed them from the IRB.

Powered by Editorial Manager® and ProduXion Manager® from Aries Systems Corporation

**Methods section of the manuscript.**<br><br>**Format for specific study types**<br><br>**Human Subject Research (involving human participants and/or tissue)**<br>• Give the name of the institutional review board or ethics committee that approved the study<br>• Include the approval number and/or a statement indicating approval of this research<br>• Indicate the form of consent obtained (written/oral) or the reason that consent was not obtained (e.g. the data were analyzed anonymously)<br><br>**Animal Research (involving vertebrate animals, embryos or tissues)**<br>• Provide the name of the Institutional Animal Care and Use Committee (IACUC) or other relevant ethics board that reviewed the study protocol, and indicate whether they approved this research or granted a formal waiver of ethical approval<br>• Include an approval number if one was obtained<br>• If the study involved *non-human primates*, add *additional details* about animal welfare and steps taken to ameliorate suffering<br>• If anesthesia, euthanasia, or any kind of animal sacrifice is part of the study, include briefly which substances and/or methods were applied<br><br>**Field Research**<br><br>Include the following details if this study involves the collection of plant, animal, or other materials from a natural setting:<br>• Field permit number<br>• Name of the institution or relevant body that granted permission	
**Data Availability**<br><br>Authors are required to make all data underlying the findings described fully available, without restriction, and from the time of publication. PLOS allows rare exceptions to address legal and ethical concerns. See the PLOS Data Policy and FAQ for detailed information.	No - some restrictions will apply

A Data Availability Statement describing where the data can be found is required at submission. Your answers to this question constitute the Data Availability Statement and **will be published in the article**, if accepted.<br><br>**Important:** Stating 'data available on request from the author' is not sufficient. If your data are only available upon request, select 'No' for the first question and explain your exceptional situation in the text box.<br><br>Do the authors confirm that all data underlying the findings described in their manuscript are fully available without restriction?	
**Describe where the data may be found in full sentences. If you are copying our sample text, replace any instances of XXX with the appropriate details.**<br><br>• If the data are **held or will be held in a public repository**, include URLs, accession numbers or DOIs. If this information will only be available after acceptance, indicate this by ticking the box below. For example: *All XXX files are available from the XXX database (accession number(s) XXX, XXX.*).<br>• If the data are all contained **within the manuscript and/or Supporting Information files**, enter the following: *All relevant data are within the manuscript and its Supporting Information files.*<br>• If neither of these applies but you are able to provide **details of access elsewhere**, with or without limitations, please do so. For example:<br><br>*Data cannot be shared publicly because of [XXX]. Data are available from the XXX Institutional Data Access / Ethics Committee (contact via XXX) for researchers who meet the criteria for access to confidential data.*<br><br>*The data underlying the results*	Data available on request from the author.

presented in the study are available from (include the name of the third party and contact information or URL).<br>• This text is appropriate if the data are owned by a third party and authors do not have permission to share the data.<br><br>* typeset	
Additional data availability information:	

Powered by Editorial Manager® and ProduXion Manager® from Aries Systems Corporation

Manuscript Click here to access/download;Manuscript;Manuscript.docx

Prompt Treatment of Fever and its Associated Factors among Under-five Children in sub-Saharan Africa: A Multilevel Analysis of Evidence from 36 Countries

Enyew Getaneh Mekonen[1]*, Belayneh Shetie Workneh[2], Tadesse Tarik Tamir[3]; Alebachew Ferede Zegeye[4]

[1]Department of Surgical Nursing, School of Nursing, College of Medicine and Health Sciences, University of Gondar, Gondar, Ethiopia; [2]Department of Emergency and Critical Care Nursing, School of Nursing, College of Medicine and Health Sciences, University of Gondar, Gondar, Ethiopia; [3]Department of Pediatrics and Child Health Nursing, School of Nursing, College of Medicine and Health Sciences, University of Gondar, Gondar, Ethiopia; [4]Department of Medical Nursing, School of Nursing, College of Medicine and Health Sciences, University of Gondar, Gondar, Ethiopia

* Corresponding author: enyewgetaneh12@gmail.com

Abstract

Background: Despite the decrease in the global under-five mortality rate, the highest rates of mortality are reported in sub-Saharan Africa. More than one-third of all deaths among under-five children are either from lower respiratory tract infections, diarrhea, or malaria. Poor treatment-seeking behavior for fever among mothers of under-five children is a big concern in sub-Saharan Africa. However, the pooled prevalence of prompt treatment of fever and its associated factors among under-five children in the region using nationally representative data is not known. Therefore, the findings of this study will inform policymakers and program managers who work on child health to design interventions to improve the timely and appropriate treatment of fever among under-five children.

Methods: Data from the recent demographic and health surveys of 36 countries in sub-Saharan Africa conducted between 2006 and 2022 were used. A total weighted sample of 71,503 living children aged under five years with a fever was included in the study. Data extracted from DHS data sets were cleaned, recorded, and analyzed using STATA/SE version 14.0 statistical software. Multilevel mixed-effects logistic regression was used to determine the factors associated with the outcome variable. Intra-class correlation coefficient, likelihood ratio test, median odds ratio, and deviance (-2LLR) values were used for model comparison and fitness. Finally, variables with a p-value <0.05 and an adjusted odds ratio with a 95% confidence interval were declared statistically significant.

Results: The pooled prevalence of prompt treatment of fever among under-five children in sub-Saharan African countries was 26.11% (95% CI: 25.79%, 26.44%). Factors like maternal education [AOR = 1.18; 95% CI (1.13, 1.25)], maternal working status [AOR = 1.34; 95% CI (1.27, 1.41)], media exposure [AOR = 1.05; 95% CI (1.01, 1.10)], household wealth index [AOR = 1.13; 95% CI (1.06, 1.19)], distance to a health facility [AOR = 1.18; 95% CI (1.13, 1.23)], healthcare decisions [AOR = 1.34; 95% CI (1.01, 1.77)], visited healthcare facility last 12 months [AOR = 1.45; 95% CI (1.38, 1.52)], antenatal care attendance [AOR = 1.79; 95% CI (1.61, 1.99)], place of delivery [AOR = 1.55; 95% CI (1.47, 1.63)], and community-level antenatal care utilization [AOR = 1.08; 95% CI (1.02,1.14)] were significantly associated with prompt treatment of fever among under-five children.

Conclusion: The pooled prevalence of prompt treatment of fever among under-five children in sub-Saharan African countries was low. Educated women, working mothers, having media exposure, rich household wealth status, perceiving distance to a health facility was not a big problem, making healthcare decisions with husband or partner, visiting healthcare facility in the

last 12 months, antenatal care attendance, health facility delivery, and high community-level antenatal care utilization increase the odds of prompt treatment of fever. Therefore, women's empowerment, information dissemination through mass media, maintaining regular visits to healthcare facilities, and strengthening health facility delivery and antenatal care services are strongly recommended.

Keywords: prompt treatment of fever, under-five children, DHS, sub-Saharan Africa, multilevel analysis

Background

Preventable and treatable causes are responsible for the deaths of an estimated five million under-five children (1). Despite the decrease in the global under-five mortality rate, the highest rates of mortality are reported in sub-Saharan Africa (SSA), which is 14 times higher than Europe and North America (1). Mortality and morbidity secondary to infection in under-five children is one of the most challenging health problems to address, as signs and symptoms are often broad and illnesses can quickly progress to severe disease (2). More than one-third of all deaths among under-five children in low- and middle-income countries (LMICs) are either from lower respiratory tract infections, diarrhea, or malaria (3). These deaths could be prevented by quick diagnosis and treatment, including antibiotics for lower respiratory infections (pneumonia), oral rehydration therapy for diarrhea, and antimalarial drugs for malaria (4).

Fever can be defined as an elevation of body temperature above 37.2°C (axillary), 37.5°C (oral), or 38°C (rectal and tympanic) (5). It is a significant public health problem among children aged 0 to 59 months in SSA, with a prevalence of 22.65% (6). Fever is the most common symptom associated with most childhood diseases and the primary cause of pediatric health facility

consultations and hospitalizations in under-five children (7, 8). In SSA, the major causes of fevers are upper and lower respiratory infections and probably viral infections, which cannot be treated using antibiotics or antimalarial drugs (6). Children from LMICs encounter around 40 or more episodes of fever by the age of five, which healthcare professionals tend to manage with antimalarial drugs or antibiotics (9).

A child presented with a complaint of fever might be diagnosed with one of the common childhood febrile illnesses, including sepsis, meningitis, herpes simplex virus, urinary tract infections, group B streptococcus disease, acute respiratory infections, enteric fever, or malaria (10, 11). Despite being the largest killer of these diseases among children under the age of five, care seeking for febrile children often occurs too late, making community-based efforts vital to increasing access to prompt treatment (12). Delayed treatment of fever cases among under-five children were 31.1% in India (13), 55.4% in Tanzania (14), and 62.1% in Nigeria (15). It is well known that fever has some potentially harmful effects if it is not managed timely and appropriately (16). Inhibited gastric emptying and impaired intestinal absorption, with accompanying anorexia and sometimes vomiting secondary to the sympathetic nervous system response to fever, are common in febrile children (17).

Studies conducted elsewhere showed that the prevalence of prompt treatment of fever was 68.9% in India (13), 37.9% in Nigeria (15), 44.6% in Tanzania (14), 42% in Mazabuka District, Zambia (18), and 57.3% in Zambia (19). Factors like child age (15), household wealth status (13, 15), mother's occupation (15), antenatal care (ANC) visits during pregnancy (15), pregnancy intention (15), number of under-five children in the household (14), distance to healthcare facility (13, 14), residence (13), child sex (19), and mother's education (19) were significantly associated with treatment of fever among under-five children.

The World Health Organization (WHO) recommends increasing efforts to achieve the Sustainable Development Goal 3.2. 1 (i.e., to end preventable deaths of under-five children by 2030 and reduce under-five mortality to at least as low as 25 per 1000 live births in every country) in SSA (1). However, poor treatment-seeking behavior for fever among mothers of under-five children is a big concern in the region and has a vast contribution to the morbidity and mortality of under-five children (14, 20). As far as our knowledge is concerned, the pooled prevalence of prompt treatment of fever and its associated factors among under-five children in SSA using nationally representative data is not known. Therefore, the findings of this study will inform policymakers and governmental and non-governmental organizations that work on child health to design interventions to improve the timely and appropriate treatment of fever among under-five children.

Methods and materials

Data sources, study design, and sampling

A cross-sectional pooled dataset using the recent DHS data from 36 SSA countries, which was conducted between 2006 and 2022, was employed. Demographic and health surveys from 36 SSA countries, including Angola (2015-16), Burkina Faso (2010), Benin (2017-18), Burundi (2016-17), Congo Democratic Republic (2013-14), Congo (2011-12), Cote d'Ivoire (2011-12), Cameroon (2022), Ethiopia (2016), Gabon (2019-21), Ghana (2014), Gambia (2019-20), Guinea (2018), Kenya (2022), Comoros (2012), Liberia (2019-20), Lesotho (2014), Madagascar (2008), Mali (2018), Malawi (2015-16), Mozambique (2011), Nigeria (2018), Niger (2019), Namibia (2013), Rwanda (2019-20), Sierra Leone (2019), Senegal (2019), Sao Tome and Principe (2008-09), Swaziland (2006-07), Chad (2014-15), Togo (2013-14), and Tanzania (2022), Uganda (2016), South Africa (2016), Zambia (2018), Zimbabwe (2015), were used. The data were appended to figure out the pooled prevalence of prompt treatment of fever and its associated factors in SSA

countries. Different datasets, including those for children, males, women, births, and households, are included in the survey for each country. For this study, the kid's record (KR) file was used. The DHS is a nationwide survey, mostly collected every five years across LMICs. It makes cross-country comparison possible as it uses standard procedures for sampling, questionnaires, data collection, cleaning, coding, and analysis (21). A total weighted sample of 71,503 living children aged under five years with a fever was included in the current study (Table 1). The DHS employs a stratified, two-stage sampling technique (22). The first stage involves the development of a sampling frame, consisting of a list of primary sampling units (PSUs) or enumeration areas (EAs), which covers the entire country and is usually developed from the latest available national census. The second stage is the systematic sampling of households listed in each cluster, or EA. Further information on the survey sampling strategies is available in the DHS guideline (23).

Variables of the study

Dependent variable: the outcome variable for this study was prompt treatment of fever (yes = 1, no = 0). Prompt treatment of fever was defined as treatment sought the same or next day following the onset of fever in under-five children as reported by mothers in the last two weeks preceding the interview (23).

Independent variables: the current study considered both individual and community-level variables. Individual level variables: mother's age (15–24 years, 25–34 years, 35–49 years), mother's education (no formal education, primary education, secondary or higher education), mother's current marital status (unmarried, married), mother's working status (not working, working), media exposure (no, yes), household wealth index (poor, middle, rich), distance to the health facility (big problem, not a big problem), health care decisions (mothers alone, with husband/partner, husband/partner alone, others), visited health facility last 12 months (no, yes),

pregnancy intention (unintended, intended), frequency of antenatal care use (none, 1–3 visits, 4+ visits), place of delivery (home, health facility), child age (0–11 months, 12–23 months, 24–59 months), child sex (male, female), and preceding birth interval (<24 months, ≥24 months). Community-level variables: place of residence (urban, rural), community-level media exposure (low, high), community-level education (low, high), community poverty level (low, high), community-level ANC utilization (low, high), and community-level health facility delivery (low, high). These factors were created by aggregating individual-level factors, as these factors were not directly available from DHS data.

Description of independent variables

Media exposure: created by combining whether a respondent reads newspapers or magazines, listens to the radio, or watches television, and is coded as "yes" if the mother was exposed to at least one of these media and "no" otherwise.

Pregnancy intention: re-categorized as intended (if the pregnancy was wanted) and unintended (incorporating both mistimed and unintended).

Community-level of media exposure: the proportion of women who had been exposed to at least one media (television, radio, or newspaper) and categorized based on the national median value as low (communities with ≤50% of women exposed) and high (communities with >50% of women exposed).

Community-level education: the proportion of women with a minimum primary level of education derived from data on respondents' level of education. Then, it was categorized using the national median value into two categories: low (communities with ≤50% of women having at least

primary education) and high (communities with >50% of women having at least primary education).

Community-level health facility delivery: the proportion of women with health facility delivery and recoded as low (communities with ≤50% of women delivered at the health facility) and high (communities with >50% of women have delivered at the health facility) community level of health facility delivery.

Community-level ANC utilization: the proportion of women with a minimum of four or more ANC visits. It is categorized using the national median value as follows: low (communities with ≤50% of women have at least four ANC visits) and high (communities with >50% of women have at least four ANC visits).

Community poverty level: an aggregated variable from household wealth status (proportion of women from poor and rich wealth status), and it was recoded as low and high community poverty level, as above.

Data management and analysis

Data extracted from the recent DHS data sets were cleaned, recorded, and analyzed using STATA/SE version 14.0 statistical software. Sample weight was employed to manage sampling errors and non-responses. Continuous variables were categorized, and categorical variables were further re-categorized. Descriptive analysis was carried out to present the data in frequencies and percentages. Both the individual and community-level variables were presented using descriptive statistics. The DHS data's variables were organized in clusters; 71,503 under-five children are nested within households, and households were nested within 1690 clusters. The assumptions of independent observations and equal variance across clusters were broken to employ the traditional

logistic regression model. This is an indication that using a sophisticated model to take into account between-cluster factors is necessary. As a result, multilevel mixed-effects logistic regression was used to determine the factors associated with prompt treatment of fever. Multilevel mixed effect logistic regression follows four models: the null model (outcome variable only), mode I (only individual-level variables), model II (only community-level variables), and model III (both individual and community-level variables). The model without independent variables (the null model) was used to check the variability of prompt treatment of fever across the cluster. The association of individual-level variables with the outcome variable (Model I) and the association of community-level variables with the outcome variable (Model II) were assessed. In the final model (Model III), the association of both individual and community-level variables was fitted simultaneously with the outcome variable (prompt treatment of fever).

The magnitude of the clustering effect and the degree to which community-level factors explain the unexplained variance of the null model were quantified by checking the intra-class correlation coefficient (ICC) and proportional change in variance (PCV). A model with the lowest deviance was selected as the best-fitted model. Finally, variables with a p-value less than 0.05 and an adjusted odds ratio (AOR) with a 95% confidence interval (CI) were described as statistically significant variables associated with prompt treatment of fever. The presence of multi-collinearity between covariates was checked by using a variance inflation factor (VIF) falling within acceptable limits of 1–10, indicating the absence of significant collinearity across independent variables.

Random-effect results

Random effects or measures of variation of the outcome variable were estimated using the median odds ratio (MOR), ICC, and PCV. The variation between clusters was measured by the ICC and

PCV. Taking clusters as a random variable, the ICC reveals that the variation of breast cancer screening between clusters is computed as ICC = VC/(VC+3.29) ×100%. The MOR is the median value of the odds ratio between the area of the highest risk and the area of the lowest risk for prompt treatment of fever when two clusters are randomly selected, using clusters as a random variable; MOR = $e^{0.95\sqrt{VC}}$. In addition, the PCV demonstrates the variation in the prevalence of prompt treatment of fever explained by factors and computed as; PCV = (Vnull-VC)/Vnull×100%; where Vnull = variance of the null model and VC = cluster level variance (24). The fixed effects were used to estimate the association between the likelihood of prompt treatment of fever and individual and community-level independent variables.

Results

Individual- and community-level characteristics of study subjects

The mean age of respondents was 28.90 ± 0.03 years, and 47.40% of them fall in the age range of 25–34 years. The majority (86.23%) of mothers were married, and 37.74% of them had no formal education. More than three-fourths (75.45%) of mothers had jobs, and 61.90% of them had media exposure. More than half (50.25%) of mothers had a poor household wealth index, and 56.56% of them reported that distance to a health facility was not a big problem. About 40.16% of mothers reported that healthcare decisions were made by husband or partners alone, and 70.67% of them visited healthcare facilities last 12 months. More than two-thirds (67.38%) of mothers had four or more ANC visits during pregnancy, and 70.24% of them wanted their pregnancy. About 68.60% of mothers gave birth at a health facility. The mean age of children was 27.08 ± 0.08 months, and 53.36% of them fall in the age range of 24–59 months. More than half (50.88%) of children were male, and the majority (85.72%) of them had a long birth interval. Only 27.34% of mothers were from urban areas, and 51.85% of them were from a community with low media exposure. More

than half (55.08%) and 53.35% of mothers were from a community with low education and high poverty levels, respectively. About 50.35% of mothers were from a communities with high ANC utilization, and 53.82% of them were from a community with high health facility delivery (Table 2).

Pooled prevalence of prompt treatment of fever

In the present study, the pooled prevalence of prompt treatment of fever among under-five children in sub-Saharan African countries was 26.11% (95% CI: 25.79%, 26.44%) (Figure 1). The result showed that the proportion of children who get prompt treatment for fever increases with increasing household wealth status, with the proportion of children who get prompt treatment for fever being lowest among children from poor household families (24.43%) and highest among rich household families (28.70%) (Figure 2). Similarly, the proportion of prompt treatment for fever was highest among mothers with secondary or higher education (29.68%) and lowest among mothers with no formal education (20.85%) (Figure 3).

Measures of variation and model fitness

A null model was used to determine whether the data supported the decision to assess randomness at the community level. Findings from the null model showed that there were significant differences in prompt treatment of fever between communities, with a variance of 0.1909212 and a P value of 0.000. The variance within clusters contributed 94.51% of the variation in prompt treatment of fever, while the variance across clusters was responsible for 5.49% of the variation. In the null model, the odds of prompt treatment of fever differed between higher- and lower-risk clusters by a factor of 1.52 times. The intra-class correlation value for Model I indicated that 1.89% of the variation in prompt treatment of fever accounts for the disparities between communities.

Then, with the null model, we used community-level variables to generate Model II. According to the ICC value from Model II, cluster variations were the basis for 5.11% of the differences in prompt treatment of fever. In the final model (model III), which attributed approximately 1.82% of the variation in the likelihood of prompt treatment of fever to both individual and community-level variables, the likelihood of prompt treatment of fever varied by 1.26 times across low and high prompt treatment of fever (Table 3).

Individual and community-level factors associated with prompt treatment of fever

In the final fitted model of multivariable multilevel logistic regression, maternal educational level, maternal occupation, media exposure, household wealth index, distance to a health facility, healthcare decisions, visited healthcare facility last 12 months, ANC visits attended during pregnancy, place of delivery, and community-level ANC utilization were significantly associated with prompt treatment of fever among under-five children in sub-Saharan African countries.

The odds of prompt treatment of fever were 1.18 and 1.15 times higher among mothers with primary and secondary education compared with mothers with no formal education, respectively [AOR = 1.18; 95% CI (1.13, 1.25)] and [AOR = 1.15; 95% CI (1.08, 1.23)]. Mothers who had jobs were 1.34 times more likely to have prompt treatment for fever compared with their counterparts [AOR = 1.34; 95% CI (1.27, 1.41)]. Mothers who listen to radio, watch television, and read newspapers were 1.05 times more likely to have prompt treatment for fever than those who had no media exposure [AOR = 1.05; 95% CI (1.01, 1.10)]. Children of mothers with rich household wealth status were 1.13 times more likely to have prompt treatment of fever compared with those from poor household wealth status [AOR = 1.13; 95% CI (1.06, 1.19)]. Mothers who reported distance to a health facility was not a big problem were 1.18 times more likely to have prompt treatment of fever than their counterparts [AOR = 1.18; 95% CI (1.13, 1.23)]. The odds of prompt

treatment of fever were 1.34 times higher among mothers who made healthcare decisions with their husband or partner than those who made decisions with someone else [AOR = 1.34; 95% CI (1.01, 1.77)].

Similarly, mothers who visited healthcare facilities in the last 12 months were 1.45 times more likely to have prompt treatment for fever compared with those who didn't [AOR = 1.45; 95% CI (1.38, 1.52)]. The frequency of ANC use was another factor significantly associated with prompt treatment of fever among under-five children. Accordingly, mothers who attended 1-3 and 4+ ANC visits were 1.57 and 1.79 times more likely to have prompt treatment of fever compared with mothers who didn't attend ANC visits during pregnancy, respectively [AOR = 1.57; 95% CI (1.41, 1.74)] and [AOR = 1.79; 95% CI (1.61, 1.99)]. The odds of prompt treatment of fever were 1.55 times higher among mothers who gave birth at a health facility compared with those who gave birth at home [AOR = 1.55; 95% CI (1.47, 1.63)]. From community-level variables, community-level ANC utilization was significantly associated with prompt treatment of fever among under-five children. Children of mothers from a community with high community-level ANC utilization were 1.08 times more likely to have prompt treatment of fever compared with those from a community with low community-level ANC utilization [AOR = 1.08; 95% CI (1.02, 1.14)] (Table 4).

Discussion

As far as the researcher's knowledge is concerned, this is the first study to determine the pooled prevalence and associated factors of prompt treatment of fever among under-five children in SSA using nationally representative DHS data. In the current study, the pooled prevalence of prompt treatment of fever among under-five children in SSA countries was 26.11% (95% CI: 25.79%, 26.44%). This finding was lower than studies conducted in Nigeria (37.9%) (15), Tanzania

(44.6%) (14), Mazabuka District, Zambia (42%) (18), Zambia (57.3%) (19), and India (37.9%) (13). The possible justification for this discrepancy might be due to differences in study setting, geographical variation, sample size, sociocultural differences, and differences in health service accessibility between countries. The difference might also be due to differences in the perceptions of caregivers of under-five children towards fever and febrile illnesses.

The study also identified individual and community-level variables associated with prompt treatment for fever. Accordingly, the odds of prompt treatment of fever were higher among educated mothers compared with mothers with no formal education. This finding was consistent with a study conducted in Zambia (19). This might be due to the fact that education plays an important role in mothers' treatment-seeking behavior for their febrile children. Studies also indicated that there is a positive association between mothers' education and their health care-seeking behavior (25, 26). This might also be explained by the fact that educated women are more likely to know the importance of seeking timely treatment for children during illnesses. Mothers who had jobs were more likely to have prompt treatment for fever compared with their counterparts. This finding was in agreement with a study conducted in Nigeria (15). This might be due to the fact that working mothers have the capability to pay for management whenever they present their febrile children to a health facility for treatment. This could also be attributed to working mothers having a higher income, being highly educated, and having adequate resources to seek prompt treatment for their febrile children, which enables them to visit healthcare facilities for fever before it gets worse. Mothers who had media exposure were more likely to have prompt treatment for fever. This finding was supported by a study conducted in Woldia, Ethiopia (27), Chad (28), and India (26). This might be due to mothers' access to radio, television, and newspapers increasing their understanding of the importance of child healthcare, and this may

describe their higher likelihood of prompt treatment of fever. Media exposure might also be associated with rich wealth status, which can also facilitate timely treatment seeking for febrile children. Media exposure is an essential instrument for health promotion and has a substantial positive influence on healthcare service utilization (29).

Children of mothers with rich household wealth status were more likely to have prompt treatment for fever compared with those with poor household wealth status. Studies conducted in Nigeria (15) and India (13) reported similar findings. This might be due to the fact that mothers with poor wealth status may not be able to afford the cost of healthcare services, and this is possible to prevent them from seeking prompt treatment for their febrile children. Mothers who reported that distance to a health facility was not a big problem were more likely to have prompt treatment for fever than their counterparts. This finding was supported by studies conducted in Tanzania (14) and India (13). This might be due to the fact that the challenges faced by mothers in accessing healthcare facilities decreased when they reported that distance to a health facility was not a big problem. A short distance to healthcare facilities may also decrease transportation costs to and from the facility which may further motivate mothers to seek early treatment for fever. The odds of prompt treatment of fever were higher among mothers who made healthcare decisions with their husband or partner than among those who made decisions with someone else. This finding was consistent with a study conducted in Nigeria (30). This might be explained by the influence of men over household resources and decision-making to simplify treatment seeking and traverse challenges in accessing care for children (31).

Likewise, mothers who visited healthcare facilities in the last 12 months were more likely to receive prompt treatment for fever compared with those who didn’t. This might be due to frequent visits to healthcare facilities, which could increase the chances of recommending timely care-

seeking behavior and increase awareness of the importance of seeking timely and appropriate medical care for febrile illnesses. Antenatal care attendance was another factor significantly associated with prompt treatment of fever, in which mothers who attended ANC visits and had high community-level ANC utilization were more likely to have prompt treatment of fever compared with their counterparts. This finding was consistent with studies conducted in Nigeria (15) and Burkina Faso (32). This might be due to fact that antenatal care attendance gives mothers the opportunity to acquire information on maternal and child health promotion (33). Accessing ANC services in LMICs is directly associated with improved birth outcomes and longer-term decreases in child mortality (34). Furthermore, the odds of prompt treatment for fever were higher among mothers who gave birth at a health facility compared with those who gave birth at home. This finding was in agreement with studies conducted in Ethiopia (35) and Nigeria (36). This might be due to the fact that mothers who gave birth at a health facility might have better awareness and trust about healthcare services, easily understand the significance of seeking healthcare services, and understand the availability of services wanted for the treatment of their febrile children. Giving birth at a health facility increases the interest of mothers in healthcare services, including healthcare professionals, and inspires them to strive for treatment when their child shows febrile disease manifestations (37).

Strengths and limitations of the study

This study has the following strengths: Firstly, the study used weighted nationally representative data from 36 sub-Saharan African countries with a large sample size. Secondly, to accommodate the hierarchical nature of the DHS data and get a reliable standard error and estimate, a multilevel analysis was employed. Thirdly, the findings of this study could be used as an input for policymakers to design proper intervention strategies both at national and regional levels, as

pooled national survey data is used. This study also had some limitations. There might be a possibility of recall and social desirability bias as the DHS survey was based on respondents' self-reports. The cross-section nature of the data couldn't enable us to show the temporal relationship between variables. Besides, we couldn't incorporate variables like knowledge and perceptions of mothers towards the treatment of fever due to the secondary nature of the data.

Conclusion

The pooled prevalence of prompt treatment of fever among under-five children in sub-Saharan African countries was low. Educated women, working mothers, having media exposure, rich household wealth status, perceiving distance to a health facility was not a big problem, making healthcare decisions with husband or partner, visiting healthcare facility in the last 12 months, ANC attendance, health facility delivery, and high community-level ANC utilization increase the odds of prompt treatment of fever. Therefore, women empowerment, information dissemination through mass media, maintaining regular visits to healthcare facilities, strengthening health facility delivery and antenatal care services, and giving prior attention to non-working mothers and those from poor household wealth status are strongly recommended to improve early treatment seeking behavior among mothers of under-five febrile children.

List of abbreviations: ANC: Antenatal Care; AOR: Adjusted Odds Ratio; CI: Confidence Interval; DHS: Demographic and Health Survey; ICC: Intra-class Correlation Coefficient; LMICs: low- and middle-income countries; MOR: Median Odds Ratio; PCV: Proportional Change in Variance; SSA: sub-Saharan Africa; VIF: Variance Inflation Factor; WHO: World Health Organization

Declarations

Ethical approval and consent to participate

Permission was granted to download and use the data from http://www.dhs.program.com before conducting the study. Ethical clearance was obtained from the Institution Review Board of the DHS Program, ICF International. The procedures for DHS public-use data sets were approved by the Institution Review Board. Identifiers for respondents, households, or sample communities were not allowed in any way, and the names of individuals or household addresses were not included in the data files. The number for each EA in the data file does not have labels to show their names or locations. There were no patients or members of the public involved since this study used a publicly available data set. As the study uses a retrospective study of archived samples, we have ensured that all data were fully anonymized before we accessed them from the IRB.

Consent for publication: not applicable.

Availability of data and materials: The data from the three SSA countries is publicly available online at (https://dhsprogram.com/data/available-datasets.cfm).

Competing interest: All authors declared that there was no competing interest.

Funding: not applicable

Author contributions: conceptualization, data curation, formal analysis, investigation, methodology, and software were done by E.G.M, B.S.W, and A.F.Z. Supervision and validation were done by E.G.M, T.T.T, and A.F.Z. Visualization and writing the original draft were done by E.G.M, B.S.W, and T.T.T. Writing, reviewing, and editing were done by E.G.M, A.F.Z, T.T.T, and B.S.W. All authors gave final approval of the version to be published; have agreed on the journal to which the article has been submitted; and agree to be accountable for all aspects of the work.

Acknowledgments: We are grateful to the DHS program for letting us use the relevant DHS data in this study.

References

1. Child mortality (under 5 years). World Health organization. 28 January 2022.

2. DISEASE CONTROL PRIORITIES. Third ed. Dean T. Jamison RN, Hellen Gelband, Susan Horton, Prabhat Jha, Ramanan Laxminarayan, Charles N. Mock, editor: The World Bank; 2016.

3. Institute for Health Metrics and Evaluation (IHME). GBD compare data visualization. 2018.

4. Children: improving survival and well-being. World Health Organization. 2020.

5. National Institute for Health and Care Excellence. Feverish Illness in Children: Assessment and Initial Management in Children Younger than 5 Years. Clinical guideline No. 160. NICE, London. 2013.

6. Paddy Ssentongo VMC, Khush Shah, Thaddeus Harbaugh, and Djibril M. Ba. Factors associated with pediatric febrile illnesses in 27 countries of Sub‑ Saharan Africa. BMC Infectious Diseases. 2023;23:391.

7. Walsh AM EH. Management of childhood fever by parents: literature review. Journal of Advanced Nursing. 2006;54(2):217–27.

8. Richardson M LM. Assessment and initial management of feverish illness in children younger than 5 years: summary of NICE guidance. BMJ 2007;334:1163–4.

9. Fink G DAV, Leslie HH, Cohen J. Antibiotic exposure among children younger than 5 years in low-income and middle-income countries: a cross-sectional study of nationally representative facility-based and household-based surveys. Lancet Infect Dis. 2020;20:179–87.

10. Animut A, Mekonnen Y, Shimelis D, Ephraim E. Febrile illnesses of different etiology among outpatients in four health centers in Northwestern Ethiopia. Japanese Journal of Infectious Diseases. 2009;62(2):107-10.

11. Arifeen SE, Bryce J, Gouws E, Baqui A, Black RE, Hoque DM, et al. Quality of care for under-fives in first-level health facilities in one district of Bangladesh. Bulletin of the World Health Organization. 2005;83:260-7.

12. Black RE, Levin C, Walker N, Chou D, Liu L, Temmerman M. Reproductive, maternal, newborn, and child health: key messages from disease control priorities 3rd edition. The Lancet. 2016;388(10061):2811-24.

13. Debsarma D, Saha J, Ghosh S. Factors associated with delay in treatment-seeking behaviour for fever cases among caregivers of under-five children in India: Evidence from the National Family Health Survey-4, 2015–16. Plos one. 2022;17(6):e0269844.

14. Kassile T, Lokina R, Mujinja P, Mmbando BP. Determinants of delay in care seeking among children under five with fever in Dodoma region, central Tanzania: a cross-sectional study. Malaria journal. 2014;13:1-10.

15. Anyasodor AE, Ahmed KY, Osuagwu UL, Mgbemena NC, Kalinna BH, Thapa S, et al. Delays in seeking treatment for fever in children under five years of age in Nigeria: Evidence from the National Demographic Health Survey. Plos one. 2023;18(12):e0295772.

16. Ca D. New concepts on the pathogenesis of fever. Rev Infect Dis. 1988;10:168-89.

17. McDougall P, Harrison M. Fever and feverish illness in children under five years. Nursing Standard. 2014;28(30).

18. Hamooya BM, Chongwe G, Dambe R, Halwiindi H. Treatment-seeking behaviour for childhood fever among caretakers of Chivuna and Magoye rural communities of Mazabuka District, Zambia: a longitudinal study. BMC public health. 2016;16(1):1-7.

19. Nawa M, Phiri KE, Sialubanje C, Hamainza B, Chiwaula J. The Prevalence and Factors Associated with Prompt Diagnosis and Treatment of Fever among Under-five Children in Zambia; Evidence from a Country-wide Cross-Sectional Survey. Medical Journal of Zambia. 2023;50(2):121-31.

20. Chibwana AI, Mathanga DP, Chinkhumba J, Campbell CH. Socio-cultural predictors of health-seeking behaviour for febrile under-five children in Mwanza-Neno district, Malawi. Malaria journal. 2009;8(1):1-8.

21. Corsi DJ, Neuman M, Finlay JE, Subramanian S. Demographic and health surveys: a profile. International journal of epidemiology. 2012;41(6):1602-13.

22. Aliaga A, Ruilin R, editors. Cluster optimal sample size for demographic and health surveys. 7th International Conference on Teaching Statistics–ICOTS; 2006.

23. Croft T, Marshall AM, Allen CK, Arnold F, Assaf S, Balian S, et al. Guide to DHS Statistics: DHS-7 (version 2). Rockville, MD: ICF. 2020.

24. Sommet N, Morselli D. Keep calm and learn multilevel logistic modeling: a simplified three-step procedure using Stata, R, mplus, and SPSS. International Review of Social Psychology. 2017;30:203-18.

25. Adedokun ST, Adekanmbi VT, Uthman OA, Lilford RJ. Contextual factors associated with health care service utilization for children with acute childhood illnesses in Nigeria. PLoS One. 2017;12(3):e0173578.

26. Chandwani H, Pandor J. Healthcare-seeking behaviors of mothers regarding their children in a tribal community of Gujarat, India. Electronic physician. 2015;7(1):990.

27. Demelash D, Mebratu W. Health care seeking behavior of mothers for common childhood illness and associated factors in Woldia town administration, Northeast Ethiopia. Fam Med Med Sci Res. 2020;10(1).

28. Budu E, Seidu A-A, Ameyaw EK, Agbaglo E, Adu C, Commey F, et al. Factors associated with healthcare seeking for childhood illnesses among mothers of children under five in Chad. Plos one. 2021;16(8):e0254885.

29. Flora JA, Maibach EW, Maccoby N. The role of media across four levels of health promotion intervention. Annual review of public health. 1989;10(1):181-201.

30. Abdulkadir MB, Abdulkadir ZA, Johnson WBR. An analysis of national data on care-seeking behaviour by parents of children with suspected pneumonia in Nigeria. South African Journal of Child Health. 2016;10(1):92-5.

31. Greenspan JA, Chebet JJ, Mpembeni R, Mosha I, Mpunga M, Winch PJ, et al. Men's roles in care seeking for maternal and newborn health: a qualitative study applying the three delays model to male involvement in Morogoro Region, Tanzania. BMC Pregnancy and Childbirth. 2019;19(1):1-12.

32. Badolo H, Bado AR, Hien H, Méda N, Susuman AS. Factors associated with mothers' health care-seeking behaviours for childhood fever in Burkina Faso: findings from repeated cross-sectional household surveys. Global Health Research and Policy. 2022;7(1):37.

33. Adedokun ST, Yaya S. Factors influencing mothers' health care seeking behaviour for their children: evidence from 31 countries in sub-Saharan Africa. BMC health services research. 2020;20(1):1-9.

34. Kuhnt J, Vollmer S. Antenatal care services and its implications for vital and health outcomes of children: evidence from 193 surveys in 69 low-income and middle-income countries. BMJ open. 2017;7(11):e017122.

35. Liyew B, Tarekegn GE, Kassew T, Tsegaye N, Asfaw MG, Tilahun AD, et al. Individual and community-level factors of treatment-seeking behaviour among caregivers with febrile children in Ethiopia: A multilevel analysis. Plos one. 2022;17(3):e0264707.

36. Adeoti IG, Cavallaro FL. Determinants of care-seeking behaviour for fever, acute respiratory infection and diarrhoea among children under five in Nigeria. Plos one. 2022;17(9):e0273901.

37. Berhane M, Yimam H, Jibat N, Zewdu M. Parents' knowledge of danger signs and health seeking behavior in newborn and young infant illness in Tiro Afeta district, Southwest Ethiopia: a community-based study. Ethiopian Journal of health sciences. 2018;28(4).

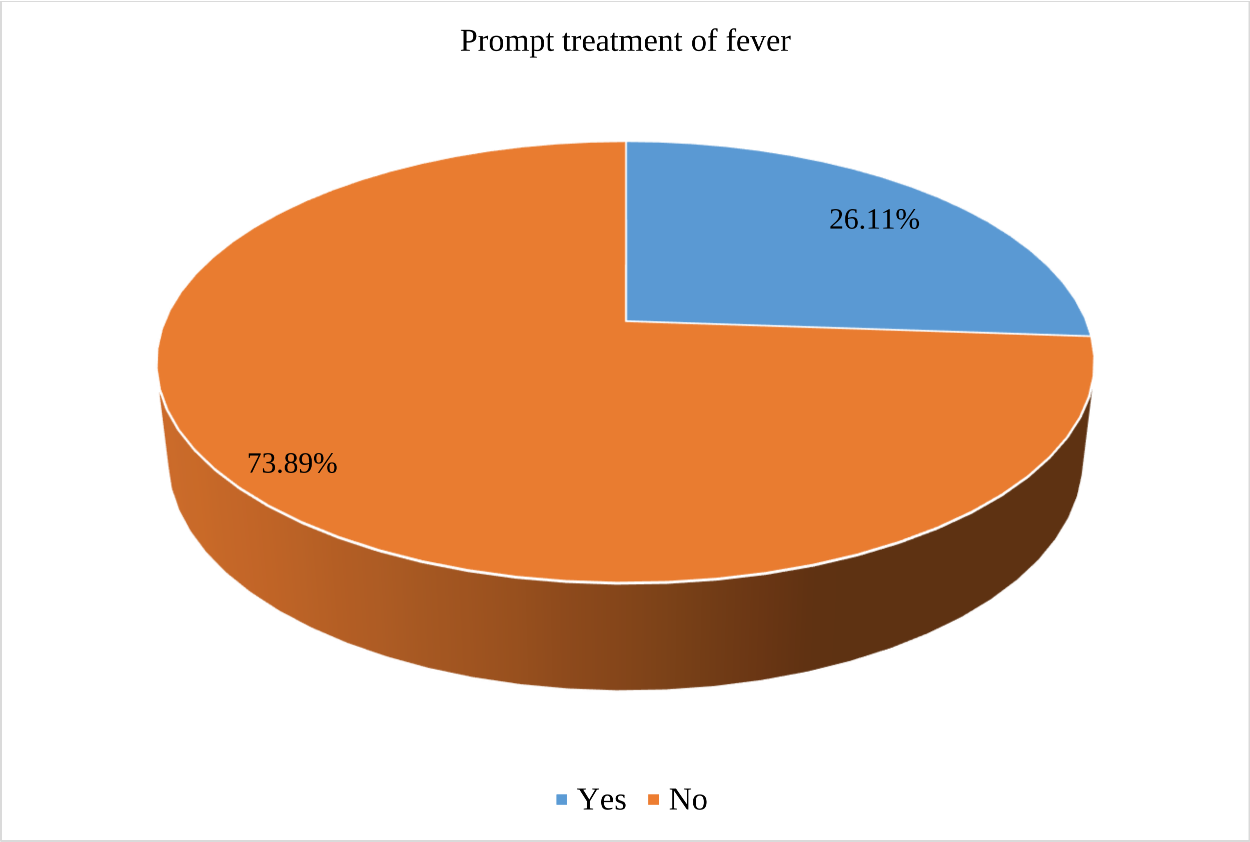

Figure 1: prevalence of prompt treatment of fever among under-five children in sub-Saharan African countries, DHS 2006-2022 (n = 71,503)

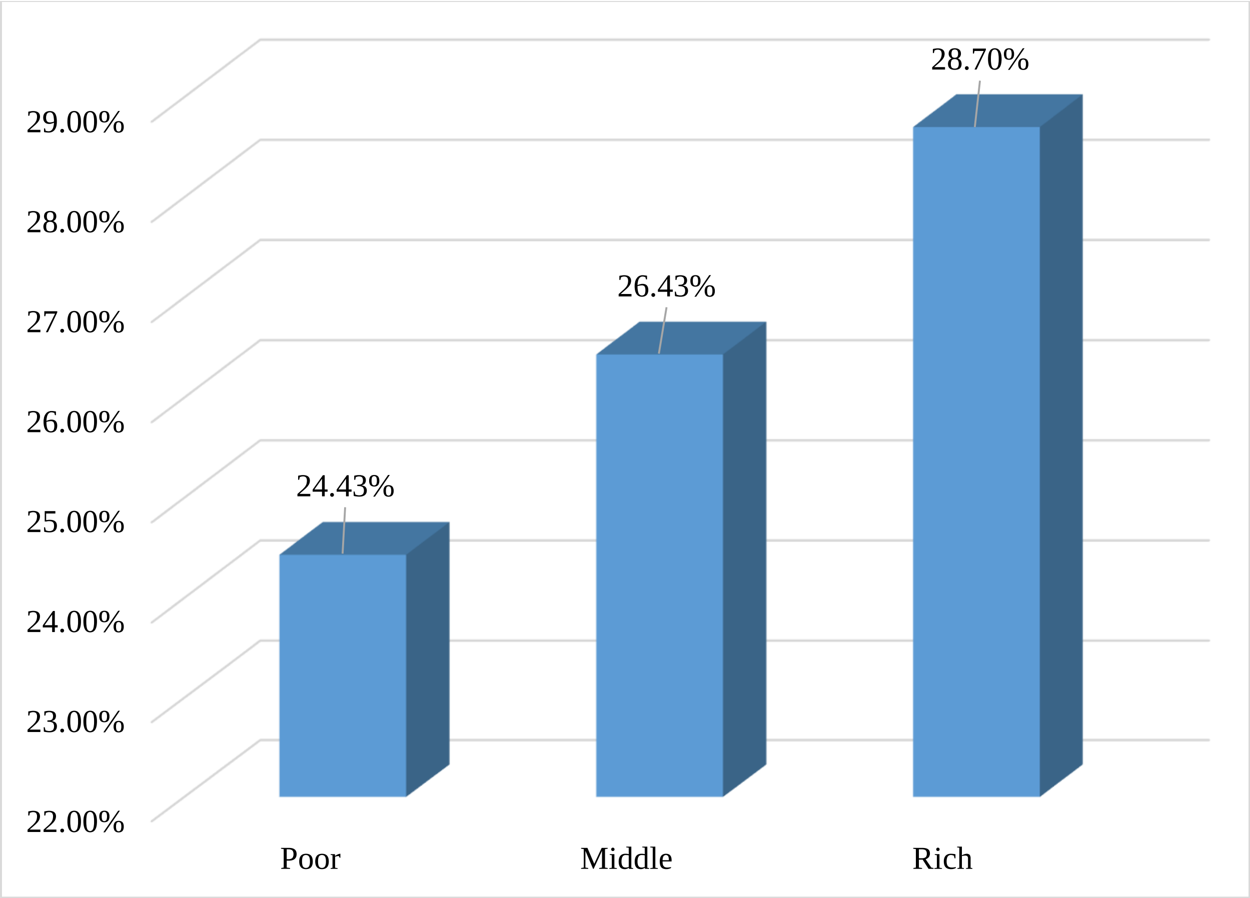

Figure 2: proportion of prompt treatment of fever by household wealth status among under-five children in sub-Saharan African countries, DHS 2006-2022 (n = 71,503)

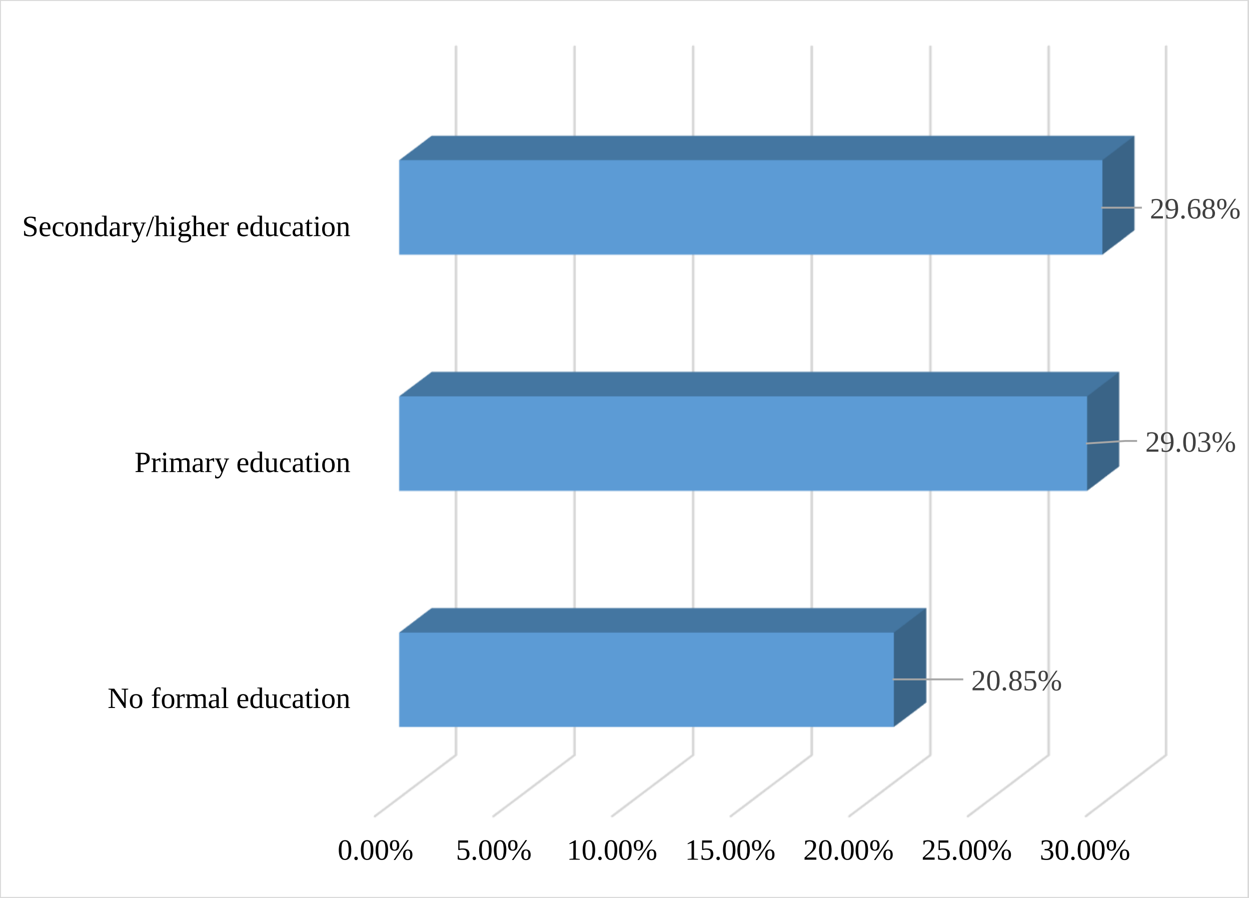

Figure 3: proportion of prompt treatment of fever by educational status among under-five children in sub-Saharan African countries, DHS 2006-2022 (n = 71,503)

Table 1: Sample size for prevalence and associated factors of prompt treatment of fever among under-five children in sub-Saharan African countries

Country	Year of survey	Weighted sample (n)	Weighted sample (%)
Angola	2015-16	1,941	2.71
Burkina Faso	2010	2,958	4.14
Benin	2017-18	2,398	3.35
Burundi	2016-17	4,528	6.33
Congo Democratic Republic	2013-14	5,157	7.21
Congo	2011-12	2,297	3.21
Cote d'Ivoire	2011-12	1,657	2.32
Cameroon	2022	1,330	1.86
Ethiopia	2016	1,350	1.89
Gabon	2019-21	1,309	1.83
Ghana	2014	822	1.15
Gambia	2019-20	1,315	1.84
Guinea	2018	1,221	1.71
Kenya	2022	3,145	4.4
Comoros	2012	635	0.89
Liberia	2019-20	1,471	2.06
Lesotho	2014	390	0.55
Madagascar	2008	1,124	1.57
Mali	2018	1,470	2.06
Malawi	2015-16	4,690	6.56
Mozambique	2011	1,313	1.84
Nigeria	2018	7,518	10.51
Niger	2019	1,563	2.19
Namibia	2013	1,138	1.59
Rwanda	2019-20	1,467	2.05
Sierra Leone	2019	1,469	2.05
Senegal	2019	922	1.29
Sao Tome and Principe	2008-09	249	0.35
Swaziland	2006-07	712	1.00
Chad	2014-15	3,539	4.95
Togo	2013-14	1,413	1.98
Tanzania	2022	1,012	1.42
Uganda	2016	4,987	6.97
South Africa	2016	647	0.90
Zambia	2018	1,549	2.17
Zimbabwe	2015	797	1.11
Total sample size		71,503	100

Table 2: Individual-and community-level characteristics of study subjects, pooled data from 36 SSA countries, DHS 2006-2022

Variables	Category	Frequency (n)	Percentage (%)
Mother's age	15–24 years	21,237	29.70
	25–34 years	33,889	47.40
	35–49 years	16,377	22.90
Mother's education	No formal education	26,986	37.74
	Primary	25,592	35.79
	Secondary/higher	18,925	26.47
Mother's current marital status	Unmarried	9,666	13.77
	Married	60,507	86.23
Mother's working status	Not working	17,552	24.55
	Working	53,951	75.45
Media exposure	No	27,194	38.10
	Yes	44,189	61.90
Household wealth index	Poor	35,931	50.25
	Middle	13,896	19.43
	Rich	21,676	30.32
Distance to a health facility	Big problem	28,224	43.44
	Not a big problem	36,750	56.56
Health care decisions	Mothers alone	10,252	14.34
	With husband/partner	21,039	29.43
	Husband/partner alone	28,719	40.16
	Others	11,493	16.07
Visited healthcare facility last 12 months	No	20,133	29.33
	Yes	48,500	70.67
Pregnancy intention	Unintended	20,457	29.76
	Intended	48,294	70.24
Frequency of antenatal care use	None	5,116	7.15
	1-3 visits	18,209	25.47
	4+ visits	48,178	67.38

Table 2 continued

Place of delivery	Home	22,450	31.40
	Health facility	49,053	68.60
Child age	0–11 months	9,955	21.39
	12–23 months	11,750	25.25
	24–59 months	24,831	53.36
Child sex	Male	36,380	50.88
	Female	35,123	49.12
Preceding birth interval	<24 months	10,208	14.28
	≥24 months	61,295	85.72
Place of residence	Urban	19,550	27.34
	Rural	51,953	72.66
Community-level media exposure	Low	37,077	51.85
	High	34,426	48.15
Community-level education	Low	39,381	55.08
	High	32,122	44.92
Community poverty level	Low	33,353	46.65
	High	38,150	53.35
Community-level of ANC utilization	Low	35,504	49.65
	High	35,999	50.35
Community-level health facility delivery	Low	33,017	46.18
	High	38,486	53.82

Table 3: Model comparison and random effect analysis for prompt treatment of fever and its associated factors in SSA countries, DHS 2006-2022 (n = 71,503)

Parameter	Null model	Model I	Model II	Model III
Variance	0.1909212	0.0632378	0.1772304	0.061124
ICC	5.49%	1.89%	5.11%	1.82%
MOR	1.52	1.27	1.49	1.26
PCV	Reference	66.88%	7.17%	67.98%
**Model fitness**				
LLR	-40731.897	-27248.049	-40663.021	-27244.342
Deviance	81463.794	54496.098	81326.042	54488.684

ICC: Intra cluster correlation; LLR: log-likelihood ratio; MOR: median odds ratio; PCV: Proportional change in variance

Table 4: Multivariable multilevel logistic regression analysis of individual and community-level factors associated with prompt treatment of fever among under-five children in SSA countries

**Variables**	**Category**	**Model I AOR (95% CI)**	**Model II AOR (95% CI)**	**Model III AOR (95% CI)**
Mother's age	15–24 years	1		1
	25–34 years	1.01(0.96,1.05)		1.01(0.95,1.05)
	35–49 years	0.99(0.94,1.06)		1.00(0.94,1.06)
Mother's education	No formal education	1		1
	Primary	1.19(1.13,1.25)*		1.18(1.13,1.25)*
	Secondary/higher	1.16(1.09,1.23)*		1.15(1.08,1.23)*
Current marital status	Unmarried	1		1
	Married	0.79(0.60,1.05)		0.79(0.60,1.05)
Mother's working status	Not working	1		1
	Working	1.34(1.27,1.41)*		1.34(1.27,1.41)*
Media exposure	No	1		1
	Yes	1.05(1.00,1.10)*		1.05(1.01,1.10)*
Household wealth index	Poor	1		1
	Middle	1.01(0.95,1.06)		1.00(0.95,1.06)
	Rich	1.13(1.07,1.19)*		1.13(1.06,1.19)*
Distance to a health facility	Big problem	1		1
	Not a big problem	1.18 (1.13,1.23)*		1.18(1.13,1.23)*
Healthcare decisions	Mothers alone	1.20(0.90,1.58)		1.19(0.90,1.58)
	With partner	1.34(1.01,1.77)*		1.34(1.01,1.77)*
	Partner alone	1.22(0.92,1.61)		1.22(0.92,1.61)
	Others	1		1
Visited health facility	No	1		1
	Yes	1.45(1.38,1.52)*		1.45(1.38,1.52)*
Pregnancy intention	Unintended	1		1
	Intended	1.02(0.98,1.07)		1.02(0.98,1.07)
Frequency of ANC use	None	1		1
	1-3 visits	1.57(1.41,1.75)*		1.57(1.41,1.74)*
	4+ visits	1.80(1.62,2.00)*		1.79(1.61,1.99)*

Table 4 continued

Place of delivery	Home	1		1
	Health facility	1.55(1.47,1.63)*		1.55(1.47,1.63)*
Child age	0–11 months	1		1
	12–23 months	1.03(0.98,1.10)		1.03(0.98,1.10)
	24–59 months	1.04(0.99,1.10)		1.04(0.99,1.10)
Child sex	Male	1.01(0.97,1.05)		1.01(0.97,1.05)
	Female	1		1
Preceding birth interval	<24 months	1		1
	≥24 months	1.02(0.96,1.08)		1.02(0.96,1.08)
Place of residence	Urban		1.11(1.07,1.16)*	1.01(0.95,1.06)
	Rural		1	1
Community media exposure	Low		1	1
	High		1.12(1.04,1.21)*	0.98(0.92,1.04)
Community-level education	Low		1	1
	High		1.30(1.21,1.40)*	1.01(0.95,1.07)
Community poverty level	Low		1.02(0.95,1.10)	0.99(0.93,1.05)
	High		1	1
Community ANC utilization	Low		1	1
	High		1.07(1.00,1.15)*	1.08(1.02,1.14)*
Community-level health facility delivery	Low		1	1
	High		0.94 (0.88,1.01)	0.97(0.91,1.03)
